# Artificial intelligence–based chatbots to enhance medication adherence among patients with non-communicable chronic diseases: Systematic review and meta-analysis

**DOI:** 10.1371/journal.pdig.0001507

**Published:** 2026-07-16

**Authors:** Siyu Chen, Yuan Fang, Liwen Ding, Phoenix K. H. Mo, Zixin Wang

**Affiliations:** Centre for Health Behaviours Research, JC School of Public Health and Primary Care, the Chinese University of Hong Kong, Hong Kong, China; Queensland Health, AUSTRALIA

## Abstract

Medication adherence remains a major public health challenge among patients with non-communicable diseases (NCDs) worldwide. Artificial intelligence (AI)–based chatbots may enhance adherence by automating reminders, education, and real-time support. This systematic review and meta-analysis evaluated the effectiveness of AI-based chatbots in improving medication adherence among patients with NCDs. This review (CRD420251151031) is reported in accordance with the PRISMA guidelines. Relevant studies were identified from PubMed, MEDLINE, Embase, Web of Science, Global Health, CINAHL, Cochrane Library, APA PsycINFO, and APA PsycArticles up to August 2025. Eligible designs included randomized controlled trials (RCTs), quasi-experimental studies, and single-arm pre–post studies. Seven studies published between 2017 and 2025 were included, of which six RCTs contributed to the meta-analysis. The pooled standardized mean difference (SMD) for medication adherence was 0.69 (95% confidence interval: 0.17 to 1.22; p = 0.01), indicating a significant medium effect with high heterogeneity (*I*^2^ = 97%). Subgroup analyses revealed greater effects for cardiovascular diseases and short-term interventions (<6 months). Daily and weekly to monthly interventions were effective, whereas on-demand or episodic interventions were not. Significant improvements were also observed when adherence was assessed using self-reported measures or pill counts. AI-based chatbots that incorporated three functions (medication reminders, education and health coaching, and real-time question-answer features) showed significant improvement in medication adherence, whereas those with dual functions did not. These findings indicate that AI-based chatbots significantly improve medication adherence in NCDs. Chatbots have the potential to supplement existing interventions to support medication adherence.

## Author summary

People living with long-term diseases such as heart disease, diabetes, or cancer often need to take medication every day, yet many struggle to stay consistent. This raises an important question of whether artificial intelligence (AI)-based chatbots, computer programs that can send reminders, answer questions, and provide support, can help people take their medicines more regularly. To do this, we reviewed and analyzed all available research studies from around the world. Overall, we found that chatbot interventions significantly improved how well people took their medications, particularly when the chatbots provided frequent reminders, educational messages, or real-time interactions. Participants reported that these systems were easy to use, convenient, and helpful in managing their daily treatment routines. AI-based chatbots can deliver support automatically and reach many people at once, and they may be especially valuable in areas with limited healthcare resources or workforce shortages. Our findings suggest that AI-based chatbots have strong potential to become an innovative tool for improving medication adherence.

## 1 Introduction

Globally, non-communicable diseases (NCDs) remain a major public health challenge, causing about 43 million deaths in 2021 and over 70% of global mortality [1]. The growing NCDs burden in low- and middle-income countries (LMICs) continues to strain health and economic systems [1,2]. Many NCDs like cardiovascular diseases, cancer, diabetes, and chronic respiratory diseases require lifelong treatment and management. Therefore, adequate adherence to NCDs treatment is essential for preventing disease progression and death. However, meta-analyses showed that the overall adherence to NCDs treatment remained inadequate, ranging from 27 to 40% for hypertension [3], 55.4% for diabetes [4], and 75% for cancer [5].

Systematic reviews have identified multiple determinants of medication non-adherence at both patient and system levels. Patient-related factors include forgetfulness, limited disease knowledge, low risk perception, concerns about side effects, complex regimens, and low self-efficacy [6,7]. System-level barriers involve limited consultation time, inadequate patient-provider communication, insufficiently tailored counselling, and lack of structured follow-up [8,9]. Together, these barriers underscore the need for sustainable, personalized interventions that deliver continuous real-world support.

A variety of interventions have been developed to improve medication adherence among patients with NCDs. Four key strategies to improve medication adherence were identified by international health authorities, including: (i) patient education and counselling to enhance knowledge, motivation, and self-management; (ii) reminder systems that track medication use and provide adherence feedback; (iii) regimen simplification, such as fixed-dose combinations; and (iv) financial incentives, including reduced co-payments or performance-based rewards [10,11]. These strategies are consistent with the Chronic Care Model, which emphasizes patient self-management support and proactive care in chronic disease management [12]. However, regimen simplification and financial incentives are not always feasible or sustainable. Most interventions focused on patient education and counselling and reminder systems. These two approaches had modest effects, which could lead to a 4.0% to 11.9% absolute increase in medication adherence [13,14]. However, most of the existing interventions providing patient education and counselling or reminders required significant input from healthcare providers, which limited their sustainability, especially in LMICs.

Technology-based approaches have emerged as scalable and sustainable strategies to improve medication adherence, overcoming the limitations of resource-intensive traditional interventions [15]. Artificial intelligence (AI)-based chatbots, computerized programs designed to simulate human conversation through text, voice, or mobile applications [16], are increasingly used in health promotion and chronic disease management [17–20]. These systems can deliver tailored reminders, educational messages, and real-time question & answer (Q&A) support. Leveraging natural language processing (NLP) and machine learning, chatbots dynamically adapt to individual needs, offering personalized, cost-effective adherence support within routine NCDs care.

Existing evidence indicates that chatbot-based interventions can enhance medication adherence across diverse NCDs, though findings remain inconsistent. For example, Arshed et al. reported significantly improved pill-count adherence among patients with hypertension receiving chatbot support compared with usual care [21], and Prakash et al. observed higher adherence among cancer patients using chatbot-assisted chemotherapy support [22]. Conversely, Ho et al. found only a marginal, non-significant difference between chatbot and control groups (63.0% vs 60.6%) [23]. These mixed results highlight the need for a systematic synthesis of global evidence on the effectiveness of chatbots in improving medication adherence among patients with NCDs. To our knowledge, no previous systematic review and meta-analysis has comprehensively addressed this question.

To address this gap, we conducted a systematic review and meta-analysis to evaluate and quantify the effectiveness of AI-based chatbot interventions in improving medication adherence among patients with NCDs worldwide. By synthesizing global evidence, this review offers key insights into the potential of chatbots to enhance adherence and inform the design of scalable digital health strategies for chronic disease management.

## 2 Materials and methods

This systematic review and meta-analysis was registered with PROSPERO (CRD420251151031) and conducted according to the PRISMA (Preferred Reporting Items for Systematic Reviews and Meta-Analyses) guidelines. The PRISMA checklist is presented in S1 Appendix.

### 2.1 Search strategy

We systematically searched PubMed, MEDLINE, Embase, Web of Science, Global Health, CINAHL, Cochrane Library, APA PsycINFO, and APA PsycArticles for studies published up to August 29, 2025, without restrictions on country or setting. The search strategy was developed according to the PICOS framework [24]: participants (patients prescribed medications for NCDs such as diabetes, cardiovascular disease, hypertension, chronic obstructive pulmonary disease, asthma, depression, or dementia); intervention (AI-based chatbots with a clear description of intervention delivery). A chatbot is defined as a computerized program that acts to replicate human interaction through text, speech, or visual forms of communication [25–27]. AI-based chatbots are those developed based on existing AI platforms or algorithms, such as machine learning, deep learning, NLP, or generative AI [28]; comparison (no limit); outcomes (quantitative measures of medication adherence, including self-report, pill count, proportion of days covered (PDC), pharmacy refill, electronic monitoring, or biological markers); and study designs (randomized controlled trials [RCTs], quasi-experimental studies, or single-arm pre–post/post-test only designs). Boolean operators (“OR” and “AND”) were applied to combine keywords and Medical Subject Headings (MeSH) terms related to AI-based chatbots, medication adherence, and NCDs. The complete search strategies for each database are presented in Tables A to I in S2 Appendix.

### 2.2 Inclusion and exclusion criteria

A detailed summary of the inclusion and exclusion criteria is provided in Table 1. To enhance methodological robustness, we excluded grey literature (e.g., policy reports, textbooks, unpublished theses, and preprints) because such sources lack consistent quality control and formal peer review. The categories of exposure and outcome indicators included in this review are presented in Box 1.

**Table 1:** Inclusion and exclusion criteria.

Parameter	Inclusion criteria	Exclusion criteria
Population (P)	• Patients prescribed medications for non-communicable chronic diseases (e.g., diabetes, cardiovascular disease, hypertension, COPD, asthma, depression, dementia).	• Studies not involving patients (e.g., laboratory simulations, clinician-only samples).
Intervention (I)	• Use of AI-based chatbot with clear description of the intervention delivery. A chatbot is defined as computerized program that acts to replicate human interaction through text, speech, or visual forms of communications. AI-based chatbot are those developed based on existing AI platforms or algorithms, such as machine learning, deep learning, natural language understanding/processing, or generative AI.	• It was unclear whether the intervention was delivered by an AI-based chatbot • The AI-based chatbot has not been evaluated by end-users yet
Comparison (C)	• None	• None
Outcomes (O)	• Quantitative measures of medication adherence (e.g., self-report, pill count, pharmacy refill, electronic monitoring, biological markers).	• Studies without measurable adherence outcomes.
Study design (S)	• Randomized controlled trials (RCTs) • Quasi-experimental studies • Single-arm pretest or posttest only studies.	• Observational studies (e.g., cross-sectional, cohort and case-control) • Reviews, narratives, commentaries, or editorials • Dissertations, preprints, government reports, newspaper articles, textbooks, book chapters, and protocols • Laboratory studies, model and framework studies, and validation studies • Grey literature (e.g., policy reports, preprints)
Language	• English language	• All other non-English languages
Availability of full text	• Full text is available and accessible	• Full text is unavailable or inaccessible
Publication period	• From database inception to August 29, 2025	• None
Study setting	• No limit	• None

**Box 1. Exposure categories and outcomes considered in the review.**

Exposure categories

- Studies that implemented AI-based chatbots to improve medication adherence in patients with NCDs.

Outcomes

- Studies that assessed medication adherence quantitatively, such as through self-report, pill counts, PDC, pharmacy refill records, electronic monitoring devices, or biological markers.

### 2.3 Quality assessment

Risk of bias was assessed using the revised tool to assess Risk of Bias in randomized trials (RoB 2) for RCTs [29], the Risk Of Bias In Non-randomized Studies-of Interventions (ROBINS-I) for quasi-experimental studies [30], and the Risk Of Bias In Non-randomized Studies-of Exposure (ROBINS-E) for pretest-posttest studies [31]. Discrepancies between the two reviewers (SC and ZW) were resolved through discussion until consensus was reached. Each study was rated as low risk, some concerns, or high risk (Tables A to C in S3 Appendix). These assessments guided evidence synthesis: studies with a high risk of bias were excluded from the meta-analysis, and sensitivity analyses were performed by sequentially removing individual studies.

### 2.4 Data extraction

A structured data extraction form was initially developed by SC and further refined in consultation with YF and ZW, drawing on the recommendations of the Cochrane Handbook for Systematic Reviews of Interventions [32]. The form was piloted on a subset of eligible studies to ensure accuracy and consistency before being applied to all included articles. Extracted information included: publication details (title, journal, and year); study characteristics (authors, country, design, blinding procedures, and randomization methods); participant information (disease condition, inclusion and exclusion criteria, sample size, and age distribution); intervention characteristics (chatbot type, duration, frequency, intensity, and other relevant features); comparator conditions (description of usual care or alternative interventions); and study outcomes (follow-up timepoints, adherence measures, effect estimates with standard deviations, standard errors, or 95% confidence intervals (CIs), and reported statistical significance). Key conclusions from each study were also recorded. The evidence was summarized in Table 2 including the relevant study characteristics, the intervention and comparator treatments, and their outcomes.

**Table 2:** Summary of findings in application of AI-based chatbot targeting medication adherence in non-communicable chronic diseases.

Author, year, (country)	Population	Study design (duration)	Sample size	Age	Chatbot type	Chatbot functions	Intervention delivery	Safety monitoring	Control group	Outcome measurement	Main findings
Labovitz 2017 (United States) [33]	Stroke patients on anticoagulants	RCT (12 weeks)	28 (15 vs 13)	55.5 ± 16.6 vs 58.3 ± 9.8	AiCure app with AI-based chatbot	Medication reminders; Symptom monitoring & self-management	Daily monitoring for 12 weeks	Real-time AI monitoring with automated alerts to clinic staff for missed doses, plus routine clinic visits with laboratory monitoring.	Standard-of-care	Pill counts, plasma levels, AI-confirmed ingestion	AI monitoring significantly improved medication adherence across pill count and plasma measures. Pill count: Mean (SD) cumulative adherence 0.972 (0.044) in intervention vs 0.906 (0.058) in control.
Horne 2022 (United States) [34]	Adults prescribed statins	RCT, 12 months	182 (89 vs 93)	63.3 ± 8.2 vs 63.1 ± 8.7	AI behavioral chatbot (CareCentra)	Medication reminders; Education & health coaching	Weekly–monthly nudges linked to refill events for 12 months	Quarterly safety assessment calls monitoring adverse events and intervention effects	Standard-of-care	Pharmacy claims data (proportion of days covered [PDC])	AI-nudge group showed higher adherence to statins vs usual care (Mean (SD): 0.742 ± 0.318 in the intervention vs. 0.639 ± 0.358 in the control; p = 0.042)
Lau-Min 2024 (United States) [35]	Gastrointestinal cancer patients on capecitabine	Single-arm study (12 weeks)	40	Median 64.5 years (IQR 49.5–72.3)	Natural language processing (NLP)-based bidirectional chatbot	Medication reminders; Real-time Q&A; Symptom monitoring & self-management	Daily reminders with weekly symptom check-ins; on-demand patient-initiated messages allowed	Daily audit of chatbot interactions with escalation of severe symptoms to oncology team	N.A.	Self-reported adherence via chatbot	Self-reported medication adherence was 71.8% (SD 29.5); chatbot recommendations >90% accurate; no serious safety concerns.
Prakash 2024 (India) [22]	Cancer patients receiving chemotherapy	Open-label RCT (6 months)	262 (131 vs 131)	Not reported	Mobile chatbot app	Medication reminders; Education & health coaching	Continuous app use with reminders for 3 months; On-demand or episodic interventions	Not reported	Standard-of-care	Medication Adherence Measure Scale (MAMS)	Mean MAMS score higher in intervention (4.95 ± 1.24) vs control (3.59 ± 0.94, p < 0.001); moderate/high adherence 71.8% vs 27.5%;
Arshed 2024 (Pakistan) [21]	Hypertension adults	Single-blinded RCT (6 months)	439 (220 vs 219)	Mean 39.5	WhatsApp-based AI intervention	Medication reminders; Real-time Q&A (WhatsApp replies, 24/7 doctor support); Education & health coaching	Daily reminders with on-demand support	24/7 physician support for medication queries and adverse effects	Standard-of-care	Self-Efficacy for Appropriate Medication Adherence Scale (SEAMS); pill count	Intervention improved adherence (SEAMS median [32], IQR [11] vs median [21], IQR [6]; P < .001); pill-count adherence (83/220, 37.2% vs 2/219, 0.9%; P < 0.001) and reduced SBP (~7 mmHg more than control).
Caballero Mateos 2025 (Spain) [36]	Type 2 diabetes patients	Multicenter RCT (6 months)	85 (44 vs 41)	53.9 ± 12.6 vs 54.3 ± 11.2	Digital Diabetes Coach (chatbot-supported)	Medication reminders; Real-time Q&A; Education & health coaching	Weekly support with optional on-demand messages (6 months)	Routine clinical care with specialist follow-up every 3 months	Standard-of-care	Morisky–Green–Levine adherence scale	Intervention: medication adherence from 58.6% to 72.4% (+13.8%, P =.01) Control: from 60% to 52% (–8%, P = .01); Also, intervention improved HbA1c, BMI, fasting glucose, and knowledge.
Ho 2025 (United States) [23]	Adults with cardiovascular conditions and refill gaps	Pragmatic RCT (12 months)	4640 (2319 vs 2321)	60.1 ± 12.7 vs 60.1 ± 12.6	Behavioral nudge chatbot	Medication reminders	Triggered messages after ≥7-day refill gaps; 12-month follow-up; on-demand or episodic interventions	Data and Safety Monitoring Board oversight; pharmacist support	Standard-of-care	PDC over 12 months (pharmacy refill data, CMS Star methodology)	Mean PDC: 63.0% (behavioral nudge + chatbot) vs. 60.6% (usual care). Adjusted differences vs. usual care: + 2.3% (0.4-4.2) (chatbot) – statistically significant before multiple comparison correction, but not after.

### 2.5 Data analysis

We performed meta-analyses using random-effects models to pool data and calculate standardized mean differences (SMDs) with 95% CIs for both RCTs and pretest-posttest designs. Hedge’s *g* was used to estimate the SMDs to remove bias due to the varying sample sizes of the included studies [37]. Statistical significance was set at p < 0.05, and SMDs values were interpreted using conventional thresholds: trivial (0.00–0.19), small (0.20–0.49), medium (0.50–0.79), or large (≥0.80) [38,39].

Sensitivity analyses were conducted by sequentially excluding individual studies. Subgroup analyses explored potential effect modifiers, including disease type (cardiovascular and cancer), intervention duration (<6 and ≥6 months), outcome measurement (self-reported, pill counts, and PDC), chatbot functions (medication reminders, symptom monitoring/self-management, education/health coaching, and real-time Q&A), and intervention intensity (daily, weekly/monthly, and on-demand/episodically).

Between-study heterogeneity was assessed using the Q test (p < 0.05 indicating significance) [40] and quantified with *I*^2^ statistics (small <25%, moderate 25–75%, large >75%) [41]. Publication bias was evaluated through funnel plot inspection and Egger’s test [42]. All analyses were conducted using Review Manager (Version 5.4; The Cochrane Collaboration, 2020) and R (Version 4.2.2; R Core Team, 2022) with the *meta* package.

## 3 Results

### 3.1 Identification of studies

**Fig 1:**
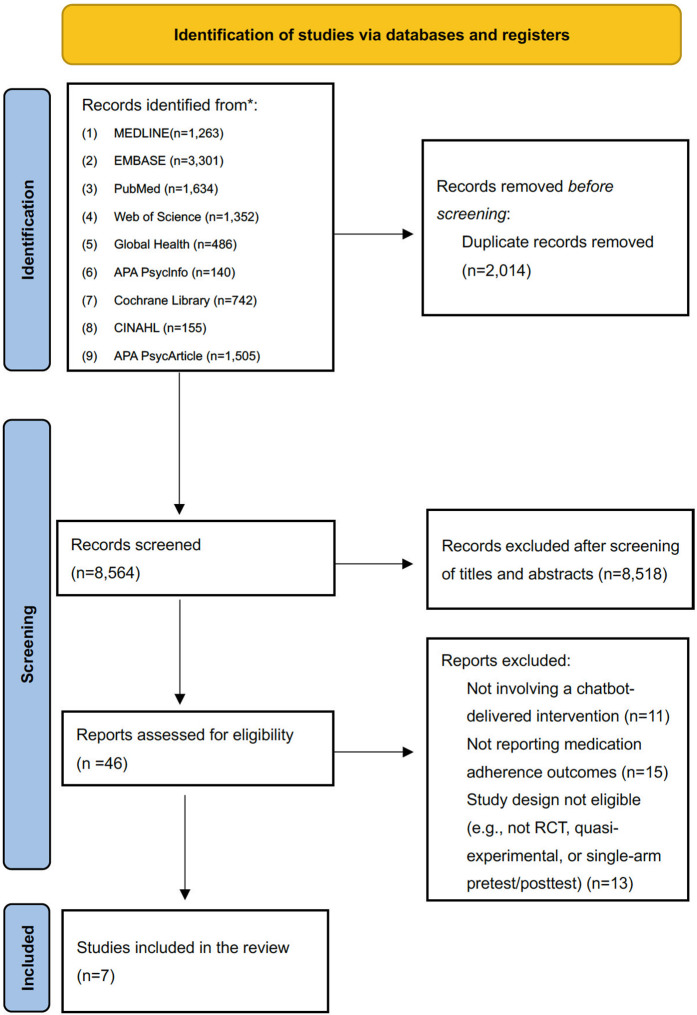
PRISMA diagram. The diagram illustrates the process of identifying, screening, and including studies in this systematic review and meta-analysis.

Of 10,578 records identified, 2,014 duplicates were removed. After screening 8,564 titles and abstracts, 46 full texts were reviewed, and 7 studies were included. Reasons for exclusion included absence of AI-based chatbot interventions (n = 11), lack of medication adherence outcomes (n = 15), and ineligible study design (n = 13). Six RCTs were eligible for meta-analysis. The study selection process is summarized in the PRISMA flow diagram (Fig 1).

### 3.2 Overview of included studies

Seven studies published between 2017 and 2025 were included in this review (Table 2) [21–23,33–36]. More than half were conducted in North America (n = 4), and the remainder were conducted in the Asia-Pacific region (n = 2) and Europe (n = 1). In terms of study design, six studies were RCTs [21–23,33,34,36] and one was a single-arm pretest-posttest study [35]. In total, 5,676 participants were enrolled, including 28 stroke patients, 182 patients prescribed statins, 302 cancer patients, 439 patients with hypertension, 85 patients with type 2 diabetes, and 4,640 patients with at least one chronic cardiovascular condition. Mean participant ages ranged from 39.5 to 64.5 years. The included studies examined adherence to anticoagulants (n = 1) [33], statins (n = 2) [23,34], chemotherapy or cancer medications (n = 2) [22,35], antihypertensive medication (n = 1) [21], and oral hypoglycemics (n = 1) [36]. All six RCTs were assessed as having a low risk of bias and were therefore retained for meta-analysis, while the single-arm study was also rated low risk. Detailed quality assessments are presented in Tables A to C in S3 Appendix.

### 3.3 Characteristics of AI-based chatbot interventions

#### 3.3.1 Functions of AI-based chatbot.

All seven studies incorporated medication reminders as a core chatbot function (n = 7). However, the format and delivery of these reminders differed across interventions. For example, Arshed et al. implemented a WhatsApp-based chatbot that delivered reminders in multiple formats, including text, voice, and graphics [21]. In contrast, Labovitz’s chatbot confirmed ingestion visually and notified clinical staff if doses were late or missed, in addition to providing medication reminders [33]. Ho et al. used a trigger-based chatbot that sent automated reminders after refill delays [23].

Among the included studies, one study included medication reminders only [23]. Three other studies combined reminders with additional functions, including symptom monitoring and self-management (n = 1) [33] and education and health coaching (n = 2) [22,34]. The remaining three studies integrated three functions: either medication reminders, symptom monitoring and self-management, and real-time Q&A support (n = 1) [35] or medication reminders, education and health coaching, and real-time Q&A support (n = 2) [21,36] (Table 2). For example, Lau-Min et al. implemented an NLP-based chatbot that provided daily medication reminders while also enabling patients to report medication errors such as “WRONG MED,” triggering system or provider follow-up [35]. Similarly, Caballero Mateos et al. integrated weekly educational sessions with real-time patient Q&A support via WhatsApp, Skype, and email [36].

#### 3.3.2 Duration, frequency and intensity of AI-based chatbot.

Intervention duration ranged from 12 weeks to 12 months, with four long-term (≥6 months) [21,23,34,36] and three short-term (<6 months) studies [22,33,35]. Regarding the intensity of intervention, three studies provided daily intervention [21,33,35]. For example, Labovitz et al. used an AI-based chatbot to confirm every pill ingestion in real time daily [33]. Two other studies provided weekly or monthly interventions [34,36]. Horne et al. used personalized chatbot weekly to monthly, typically timed around prescription refills [34]. The rest of them provided on-demand or episodic interventions [22,23]. For example, Ho et al. delivered chatbot messages only when a patient experienced a ≥ 7-day refill gap, with up to five messages per episode [23].

#### 3.3.3 Measurements of medication adherence.

Outcome assessment methods varied across the included studies. Three studies relied solely on self-reported adherence outcomes, including the Medication Adherence Measure Scale [22], self-reported adherence via chatbot responses [35], and the 4-item Morisky-Green-Levine Questionnaire [36]. For example, Lau-Min et al. measured adherence based on daily self-reported confirmations (“TAKEN”) within the chatbot platform, achieving a mean adherence rate of 71.8% [35]. Three studies used pill counts [33] or pharmacy refill data calculating the PDC [23,34]. One study [21] employed both self-reported outcome (e.g., adherence questionnaires) and pill-count verification to provide a more comprehensive evaluation of adherence.

#### 3.3.4 Control groups.

All six experimental studies used standard-of-care control groups to evaluate the effectiveness of AI-based chatbots [21–23,33,34,36]. The control group did not have access to the chatbot during the intervention period.

### 3.4 Key outcomes

#### 3.4.1 Medication adherence.

**Fig 2:**
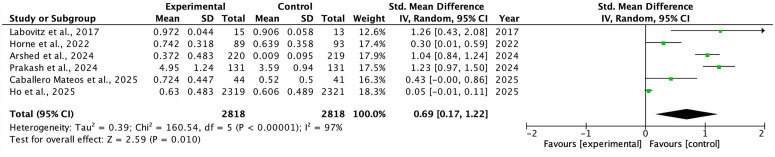
Forest plot among included studies. The figure shows studies assessing the effect of AI-based chatbot interventions on medication adherence.

Our meta-analysis synthesized evidence from six RCTs evaluating AI-based chatbot interventions for medication adherence in patients with NCDs (Table 3, Fig 2). Overall, AI-based chatbots significantly improved medication adherence compared with standard-of-care (intervention: n = 2,818; control: n = 2,818), with a pooled SMD of 0.69 (95% CI: 0.17 to 1.22, p = 0.01). Considerable heterogeneity was observed (*I*^2^ = 97%, p < 0.001).

**Table 3:** Summary of meta-analysis and sub-group analysis among included studies.

Group/subgroup	k	Effect size, heterogeneity and subgroup analysis				Publication bias by Egger’s test	
		SMD	[95% CI]	*I* ^*2*^	*P*_subgroup_	Beta (SE)	Egger’s test (p)
**Medication adherence**	6	0.69	[0.17, 1.22] *	97%**		5.43 (2.50)	0.10
Disease type					0.03*		
CVD	5	0.57	[0.05, 1.09] *	96%**		4.29 (2.62)	0.20
Cancer	1	1.23	[0.97, 1.50] **	N.A.		N.A.	N.A.
							
Duration of intervention					0.01*		
Short-term (<6 months)	2	1.23	[0.98, 1.49] **	0%		N.A.	N.A.
Long-term (>=6 months)	4	0.45	[-0.10, 1.00]	97%**		4.92 (3.82)	0.33
							
Intensity of intervention					<0.001**		
Daily	2	1.05	[0.86, 1.25] **	0%		N.A.	N.A.
Weekly/monthly	2	0.34	[0.10, 0.58] **	0%		N.A.	N.A.
On-demand/episodically	2	0.63	[-0.53, 1.79]	99%**		N.A.	N.A.
Outcome measurements					<0.001**		
Self-reported	3	1.14	[0.52, 1.75] **	93%**		-11.52 (2.24)	0.12
Pill counts	2	1.05	[0.86, 1.25] **	0%		N.A.	N.A.
Proportion of days covered	2	0.13	[-0.10, 0.37]	64%		N.A.	N.A.
Chatbot function					0.001**		
Medication reminders only	1	0.05	[-0.01, 0.11]	N.A.		N.A.	N.A.
Medication reminders + symptom monitoring & self-management	1	1.26	[0.43, 2.08] **	N.A.		N.A.	N.A.
Medication reminders + education & health coaching	2	0.77	[-0.14, 1.68]	95%**		N.A.	N.A.
Medication reminders + education & health coaching + real-time Q&A	2	0.76	[0.17, 1.36] *	84%**		N.A.	N.A.

†*p* < 0.10, *p < 0.05, ** *p* < 0.01.

SMD: Standardized mean difference comparing the effectiveness of AI-based chatbot interventions with control/usual-care interventions; SMD values were categorized as trivial (0.00–0.19), small (0.20–0.49), medium (0.50–0.79), and large (≥0.80).

*I*^*2*^: *I*^*2*^ statistic quantified the degree of heterogeneity, categorized as small (<25%), medium (25%–75%), or large (>75%).

Egger’s test: Assessed the presence of publication bias by testing the significance of funnel plot asymmetry.

CVD: Cardiovascular diseases.

Subgroup analyses were conducted by stratification of disease type, the interventions significantly improved adherence among patients with cardiovascular diseases (pooled SMD = 0.57, 95% CI: 0.05 to 1.09, p = 0.03; *I*^2^ = 96%, p < 0.001), while a single study in cancer patients reported a large positive effect (SMD = 1.23, 95% CI: 0.97 to 1.50), though pooling was not feasible (Fig A in S4 Appendix). Intervention duration appeared to influence outcomes: short-term interventions (<6 months) were consistently effective (pooled SMD = 1.23, 95% CI: 0.98 to 1.49, p < 0.001; *I*^2^ = 0%, p = 0.95), whereas longer interventions (≥6 months) did not show significant effects (pooled SMD = 0.45, 95% CI: –0.10 to 1.00, p = 0.11; *I*^2^ = 97%, p < 0.001) (Fig B in S4 Appendix).

Intensity of delivery was also considered for subgroup analysis. Daily interventions produced large and consistent benefits (pooled SMD = 1.05, 95% CI: 0.86 to 1.25, p < 0.001; *I*^2^ = 0%, p = 0.61). Weekly or monthly interventions yielded modest but significant improvements (pooled SMD = 0.34, 95% CI: 0.10 to 0.58, p = 0.006; *I*^2^ = 0%, p = 0.64). By contrast, on-demand or episodic interventions did not significantly improve adherence (pooled SMD = 0.63, 95% CI: –0.53 to 1.79, p = 0.28; *I*^2^ = 99%, p < 0.001) (Fig C in S4 Appendix).

Differences were also observed depending on how adherence was measured. Interventions were effective when adherence was assessed through self-reports (pooled SMD = 1.14, 95% CI: 0.52 to 1.75, p < 0.001; *I*^2^ = 93%, p < 0.001) (Fig D in S4 Appendix) and pill counts (pooled SMD = 1.05, 95% CI: 0.86 to 1.25, p < 0.001; *I*^2^ = 0%, p = 0.61) (Fig E in S4 Appendix). Interventions that integrated three functions (medication reminders, education and health coaching, and real-time Q&A support) demonstrated significant benefits (pooled SMD = 0.76, 95% CI: 0.17 to 1.36, p = 0.01; *I*^2^ = 84%, p = 0.01), while those offering two functions did not show significant improvements (Fig F in S4 Appendix). A single study that provided medication reminders alone, and another combining medication reminders with symptom monitoring and self-management, were not included in pooling.

One additional non-experimental study, which was excluded from the meta-analysis [35], evaluated an NLP-based chatbot that provided daily reminders and weekly symptom check-ins. This study reported self-reported adherence of 71.8% (SD = 29.5) and no serious safety concerns.

#### 3.4.2 Publication bias.

Publication bias was assessed using funnel plots and Egger’s test (Table 3, Fig A in S5 Appendix). Egger’s test indicated no statistically significant evidence of publication bias for studies evaluating AI-based chatbot interventions to improve medication adherence (p = 0.10). Similarly, no significant publication bias was observed across subgroup analyses (p = 0.12 to 0.33).

#### 3.4.3 Sensitivity analysis.

Sensitivity analyses demonstrated that the pooled effect estimates remained stable when each study was excluded sequentially (Table 4). Excluding study [33] yielded a pooled SMD of 0.61 (95% CI: 0.05 to 1.17, p = 0.03; *I*^2^ = 97%, p < 0.001). Excluding study [34] resulted in a pooled SMD of 0.78 (95% CI: 0.14 to 1.41, p = 0.02; *I*^2^ = 97%, p < 0.001). Excluding study [22] produced a pooled SMD of 0.57 (95% CI: 0.05 to 1.09, p = 0.03; *I*^2^ = 96%, p < 0.001). Excluding study [21] gave a pooled SMD of 0.61 (95% CI: 0.07 to 1.16, p = 0.03; *I*^2^ = 95%, p < 0.001). Excluding study [36] resulted in a pooled SMD of 0.75 (95% CI: 0.15 to 1.34, p = 0.01; *I*^2^ = 97%, p < 0.001). Finally, excluding study [23] yielded a pooled SMD of 0.83 (95% CI: 0.43 to 1.22, p < 0.001; *I*^2^ = 86%, p < 0.001). Across all scenarios, Egger’s test showed no statistically significant evidence of publication bias (p = 0.11 to 0.71), indicating that no single study disproportionately influenced the overall results.

**Table 4:** Summary of sensitivity analyses among included studies.

Group	The study was removed	k	Effect size, heterogeneity and subgroup analysis			Publication bias by Egger’s test	
			SMD	[95% CI]	*I* ^*2*^	Beta (SE)	Egger’s test (p)
Medication adherence	Labovitz et al., 2017	5	0.61	[0.05, 1.17] *	97%**	6.37 (3.20)	0.14
Medication adherence	Horne et al., 2022	5	0.78	[0.14, 1.41] *	97%**	6.28 (2.92)	0.12
Medication adherence	Prakash et al., 2024	5	0.57	[0.05, 1.09] *	96%**	4.29 (2.62)	0.20
Medication adherence	Arshed et al., 2024	5	0.61	[0.07, 1.16] *	95%**	4.23 (2.17)	0.15
Medication adherence	Caballero Mateos et al., 2025	5	0.75	[0.15, 1.34] *	97%**	6.52 (2.95)	0.11
Medication adherence	Ho et al., 2025	5	0.83	[0.43, 1.22] **	86%**	-1.49 (3.58)	0.71

†*p* < 0.10, *p < 0.05, ** *p* < 0.01. SMD: Standardized mean difference comparing the effectiveness of AI-based chatbot interventions with control/usual-care interventions; SMD values were categorized as trivial (0.00–0.19), small (0.20–0.49), medium (0.50–0.79), and large (≥0.80).

*I*^*2*^: *I*^*2*^ statistic quantified the degree of heterogeneity, categorized as small (<25%), medium (25%–75%), or large (>75%).

Egger’s test: Assessed the presence of publication bias by testing the significance of funnel plot asymmetry.

### 3.5 Engagement with AI-based chatbot interventions

Participants in the included studies expressed positive attitudes toward chatbot use, reporting that the systems were convenient, easy to use, and supportive in promoting medication adherence. In Horne et al. (2022), participants in the chatbot group described the messages as motivating and personalized, with adherence data from pharmacy claims confirming improved statin use compared to usual care [34]. Similarly, Lau-Min et al. (2024) found that participants using a chatbot for daily reminders and symptom check-ins reported self-reported adherence of 71.8%, with over 90% of chatbot recommendations being accurate, reflecting high engagement [35]. Moreover, Arshed et al. (2024) demonstrated that patients actively interacted with the WhatsApp-based chatbot, resulting in significantly higher medication adherence scores (SEAMS), with qualitative feedback underscoring the convenience of multimedia messages and 24/7 accessibility [21].

## 4 Discussion

Medication adherence remains a major challenge in chronic disease management. The implementation of traditional interventions (e.g., in-person counselling and reminders) is resource demanding. AI-based chatbots may be a cost-effective solution to supplement existing interventions to support medication adherence. This review provides the first quantitative synthesis demonstrating that AI-based chatbots significantly improve medication adherence in NCDs.

Our review synthesized evidence from seven studies, including six in the meta-analysis, to evaluate the effectiveness of AI-based chatbot interventions for medication adherence. The pooled analysis showed a significant medium effect size (SMD = 0.69), indicating that chatbots substantially improved adherence. Several reasons may explain this effect. Advances in AI enable continuous monitoring and feedback, sustaining patient engagement and long-term adherence [43]. Consistent with this, our study found that participants reported high satisfaction with chatbot use, describing the systems as convenient, user-friendly, and supportive, with more than 90% of responses rated as accurate and relevant. Furthermore, synchronous or near-synchronous communication, such as automated reminders and real-time Q&A, facilitates timely feedback and behavioral reinforcement, reducing non-adherence [44]. Finally, the mobile accessibility of chatbots allows patients to receive support anytime and anywhere, overcoming barriers related to clinic-based counselling [26].

Subgroup analyses identified several factors influencing the effectiveness of chatbot interventions in medication adherence. Although short-term interventions (<6 months) were more effective than long-term ones, this finding should be interpreted cautiously, as outcome measurement time points differed across studies. Daily and weekly to monthly interventions improved adherence, whereas on-demand or episodic interventions were less effective. Medication adherence requires long-term changes in habits. On-demand or episodic interventions may lead to temporal improvements but are less likely to facilitate sustained behavioral changes [45,46]. In addition, barriers to medication adherence are likely to change during treatment. On-demand or episodic interventions may not be able to address these changing circumstances over time [47]. Our findings highlight the importance of structured, regular interaction to sustain behavior change [48,49]. Regarding outcome assessment, both self-reported and objectively measured adherence (e.g., pill counts) are valuable [50]. In our review, interventions using self-reported measures and pill counts demonstrated significant improvements in adherence. Self-reported medication adherence is a practical way to measure adherence because of its low cost and potential to be easily implemented into the clinical workflow. Evidence showed that self-reported adherence was predictive of clinical outcomes [50]. Different tools were used by the included studies to measure self-reported adherence (e.g., Medication Adherence Measure Scale [MAMS], Morisky Green-Levine Medication Adherence Questionnaire [MGL MAQ], or self-constructed tool). Selecting appropriate adherence measurement tools in clinical research and practice is important to accurately capture patient compliance and optimize therapeutic effectiveness. Future studies may consider using validated, standardized, and widely used tools (e.g., 8-item Morisky Medication Adherence Scale [MMAS-8]) rather than self-constructed or unvalidated measurement. However, they remain vulnerable to recall and social desirability bias. Pill counts are commonly used by included studies, which provide a more objective assessment but may still overestimate adherence if patients remove medication without ingestion [51]. For chronic and long-term medication, it is recommended to adopt PDC as a standardized objective measurement [52]. Combining self-reported and objective measures may therefore offer a more comprehensive and accurate evaluation of adherence behavior in future studies [50]. Integrating three chatbot functions (medication reminders, education and health coaching, and real-time Q&A) demonstrated significant effects, whereas dual-function interventions did not. These findings suggest that comprehensive, multi-component chatbot designs may be more effective in improving medication adherence than simpler-function systems.

Substantial heterogeneity was observed across the included studies. Simpler chatbots primarily delivered medication reminders, whereas more advanced systems incorporated educational or counselling content and real-time Q&A features. Studies also differed in adherence measures, disease types, and participant characteristics, which likely contributed to the variability in effect sizes. Sensitivity analyses did not eliminate this heterogeneity, suggesting that these differences were systematic rather than random. To facilitate evidence synthesis, future research may consider using validated and standardized self-reported (e.g., MMAS-8) and objective adherence measurements (e.g., PDC). In addition, more standardized descriptions about which key strategies to improve medication adherence (e.g., patient education and counselling, reminder system) covered by the chatbot, and the type of chatbot functionality would be helpful. For example, the conversation mechanism (rule-based, AI-powered, or hybrid), exact functions (information dissemination, question and answer), and whether human agents are involved (e.g., routing complex issues to human agents).

Despite their potential, AI-based chatbots may face an important challenge in improving medication adherence among patients with NCDs. Most included chatbots appeared to rely on predefined workflows or rule-based components, and unable to address complex or unanticipated questions without human input. Future research may consider exploring chatbot’s bias in measuring or monitoring medication adherence, due to language or technology employed by the chatbot, or health literacy of the users. Hybrid AI-based chatbots that combine rule-based workflow with large language models, such as ChatGPT, may deliver more adaptive support [53]. Future studies may also consider reviewing the human-chatbot interaction and collecting feedback from users regularly to identity areas where the chatbot is underperforming and to improve its performance. A co-design approach, which has been adopted to develop workflow and knowledge bases of some health promotion chatbots, may enhance community capacity building and ensure the chatbots meet users’ needs [54,55]. These approaches could enhance user engagement, improve adherence outcomes, and hence reduce provider burdens, ultimately improving the sustainability of chronic disease management. Five of the seven included studies reported clinical safety mechanisms, including automated alerts sent by the chatbots to designated clinical staff, safety assessment calls, access to physician support, and regular auditing of chatbot-human interactions. Another study had a Data and Safety Monitoring Board to oversee the interventions. These findings highlight the importance of clinical safety mechanisms in chatbot-delivered interventions. Future research may explore the feasibility of hybrid AI–human oversight models to ensure patient safety in chatbot-based interventions.

This review has several strengths. Rigorous and standardized methodologies were applied in accordance with PRISMA and Cochrane guidelines. A comprehensive multi-database search, systematic risk-of-bias assessment, and detailed subgroup and sensitivity analyses enhanced the robustness of the findings. No publication bias was detected, and sensitivity analyses confirmed the stability of pooled effect estimates. In addition, the inclusion of implementation-related metrics provided valuable insights into the feasibility and real-world applicability of AI-based chatbot interventions for improving medication adherence.

This study has several limitations. First, the small number of included studies and high level of heterogeneity limit the generalizability of the pooled estimates; therefore, the findings should be interpreted as preliminary evidence of the effectiveness of AI-based chatbot interventions in improving medication adherence among patients with NCDs. Second, variations in study design and outcome measures may have introduced reporting bias. Third, the predominance of studies from North America limits the applicability of findings to other settings, particularly LMICs where digital access and literacy differ. Future studies should also explore how to address inequalities to access chatbot or other AI-powered medication adherence support due to technology access or digital health literacy, especially in rural and remote settings. In addition, most interventions targeted cardiovascular diseases. Evidence for cancer and other NCDs was limited, underscoring the need for further research. Finally, reliance on self-reported adherence measures in several studies raises concerns about recall and social desirability biases.

In conclusion, this systematic review and meta-analysis demonstrate that AI-based chatbot interventions significantly improve medication adherence among patients with NCDs compared with standard-of-care. When effectively implemented, chatbots have the potential to supplement existing interventions to support medication adherence.

## Supporting information

S1 Appendix The PRISMA checklist. (DOCX)

S2 Appendix Search strategy for selected databases. (DOCX)

S3 Appendix Assessments of reporting quality in included studies. (DOCX)

S4 Appendix Forest plots for sub-group analyses. (OCX)

S5 Appendix Funnel plots for meta-analyses. (DOCX)
