# Supplementary material for: Artificial intelligence–based chatbots to enhance medication adherence among patients with non-communicable chronic diseases: Systematic review and meta-analysis
Source: PLOS Digit Health. 2026 Jul 16;5(7):e0001507. doi: 10.1371/journal.pdig.0001507 (PMC13375028; doi:10.1371/journal.pdig.0001507)
Supplement: S2 Appendix — (DOCX) [file pdig.0001507.s002.docx]

**S2 Appendix. Search strategies for selected databases**

Table A. Search strategies (MEDLINE)

| Steps | Search terms | Results (August 29, 2025) |
| --- | --- | --- |
| 1 | “artificial intelligence” OR AI OR “AI agent*” OR “artificial agent*” OR “assistance technolog*” OR “bot” OR “chat bot*” OR chatbot* OR chatterbot* OR chatgpt OR “ChatGPT” OR “GPT” OR “conversational agent*” OR “conversational AI” OR “conversational assistant*” OR “voice agent*” OR “conversational bot*” OR “conversational interface*” OR “conversational system*” OR “dialog system*” OR “dialogue agent*” OR “digital assistant*” OR “digital coach” OR “virtual coach” OR “embodied conversational agent*” OR “large language model*” OR LLM | 116,837 |
| 2 | adheren* OR complian* OR persist* OR concordan* OR continu* OR dropout* | 2,175,218 |
| 3 | medicat* OR pharma* OR drug* OR agent* OR therap* | 12,043,086 |
| 4 | “chronic disease*” OR diabet* OR “chronic lung disease*” OR “interstitial lung disease*” OR “pulmonary fibros*” OR COPD OR “chronic obstructive pulmonary disease*” OR asthm* OR “cardiovascular disease*” OR angina* OR hypertens* OR stroke* OR “cerebrovascular accident*” OR “heart disease*” OR “myocardial infarct*” OR arrhythm* OR “peripheral arter* disease*” OR “mental health” OR depress* OR anxiet* OR Parkinson* OR dementia* | 15,418,425 |
| 5 | 1 AND 2 AND 3 AND 4 | 1,263 |

Table B. Search strategies (Embase)

| Steps | Search terms | Results (August 29, 2025) |
| --- | --- | --- |
| 1 | “artificial intelligence” OR AI OR “AI agent*” OR “artificial agent*” OR “assistance technolog*” OR “bot” OR “chat bot*” OR chatbot* OR chatterbot* OR chatgpt OR “ChatGPT” OR “GPT” OR “conversational agent*” OR “conversational AI” OR “conversational assistant*” OR “voice agent*” OR “conversational bot*” OR “conversational interface*” OR “conversational system*” OR “dialog system*” OR “dialogue agent*” OR “digital assistant*” OR “digital coach” OR “virtual coach” OR “embodied conversational agent*” OR “large language model*” OR LLM | 221,139 |
| 2 | adheren* OR complian* OR persist* OR concordan* OR continu* OR dropout* | 3,799,110 |
| 3 | medicat* OR pharma* OR drug* OR agent* OR therap* | 20,539,809 |
| 4 | “chronic disease*” OR diabet* OR “chronic lung disease*” OR “interstitial lung disease*” OR “pulmonary fibros*” OR COPD OR “chronic obstructive pulmonary disease*” OR asthm* OR “cardiovascular disease*” OR angina* OR hypertens* OR stroke* OR “cerebrovascular accident*” OR “heart disease*” OR “myocardial infarct*” OR arrhythm* OR “peripheral arter* disease*” OR “mental health” OR depress* OR anxiet* OR Parkinson* OR dementia* | 25,506,832 |
| 5 | 1 AND 2 AND 3 AND 4 | 3,301 |

Table C. Search strategies (Web of Science)

| Steps | Search terms | Results (August 29, 2025) |
| --- | --- | --- |
| 1 | “artificial intelligence” OR AI OR “AI agent*” OR “artificial agent*” OR “assistance technolog*” OR “bot” OR “chat bot*” OR chatbot* OR chatterbot* OR chatgpt OR “ChatGPT” OR “GPT” OR “conversational agent*” OR “conversational AI” OR “conversational assistant*” OR “voice agent*” OR “conversational bot*” OR “conversational interface*” OR “conversational system*” OR “dialog system*” OR “dialogue agent*” OR “digital assistant*” OR “digital coach” OR “virtual coach” OR “embodied conversational agent*” OR “large language model*” OR LLM | 459,334 |
| 2 | adheren* OR complian* OR persist* OR concordan* OR continu* OR dropout* | 4,817,618 |
| 3 | medicat* OR pharma* OR drug* OR agent* OR therap* | 8,781,092 |
| 4 | “chronic disease*” OR diabet* OR “chronic lung disease*” OR “interstitial lung disease*” OR “pulmonary fibros*” OR COPD OR “chronic obstructive pulmonary disease*” OR asthm* OR “cardiovascular disease*” OR angina* OR hypertens* OR stroke* OR “cerebrovascular accident*” OR “heart disease*” OR “myocardial infarct*” OR arrhythm* OR “peripheral arter* disease*” OR “mental health” OR depress* OR anxiet* OR Parkinson* OR dementia* | 5,198,302 |
| 5 | 1 AND 2 AND 3 AND 4 | 1,352 |

Table D. Search strategies (Global Health)

| Steps | Search terms | Results (August 29, 2025) |
| --- | --- | --- |
| 1 | “artificial intelligence” OR AI OR “AI agent*” OR “artificial agent*” OR “assistance technolog*” OR “bot” OR “chat bot*” OR chatbot* OR chatterbot* OR chatgpt OR “ChatGPT” OR “GPT” OR “conversational agent*” OR “conversational AI” OR “conversational assistant*” OR “voice agent*” OR “conversational bot*” OR “conversational interface*” OR “conversational system*” OR “dialog system*” OR “dialogue agent*” OR “digital assistant*” OR “digital coach” OR “virtual coach” OR “embodied conversational agent*” OR “large language model*” OR LLM | 41,853 |
| 2 | adheren* OR complian* OR persist* OR concordan* OR continu* OR dropout* | 472,995 |
| 3 | medicat* OR pharma* OR drug* OR agent* OR therap* | 2,523,177 |
| 4 | “chronic disease*” OR diabet* OR “chronic lung disease*” OR “interstitial lung disease*” OR “pulmonary fibros*” OR COPD OR “chronic obstructive pulmonary disease*” OR asthm* OR “cardiovascular disease*” OR angina* OR hypertens* OR stroke* OR “cerebrovascular accident*” OR “heart disease*” OR “myocardial infarct*” OR arrhythm* OR “peripheral arter* disease*” OR “mental health” OR depress* OR anxiet* OR Parkinson* OR dementia* | 3,033,684 |
| 5 | 1 AND 2 AND 3 AND 4 | 486 |

Table E. Search strategies (APA PsycINFO)

| Steps | Search terms | Results (August 29, 2025) |
| --- | --- | --- |
| 1 | “artificial intelligence” OR AI OR “AI agent*” OR “artificial agent*” OR “assistance technolog*” OR “bot” OR “chat bot*” OR chatbot* OR chatterbot* OR chatgpt OR “ChatGPT” OR “GPT” OR “conversational agent*” OR “conversational AI” OR “conversational assistant*” OR “voice agent*” OR “conversational bot*” OR “conversational interface*” OR “conversational system*” OR “dialog system*” OR “dialogue agent*” OR “digital assistant*” OR “digital coach” OR “virtual coach” OR “embodied conversational agent*” OR “large language model*” OR LLM | 29,249 |
| 2 | adheren* OR complian* OR persist* OR concordan* OR continu* OR dropout* | 542,735 |
| 3 | medicat* OR pharma* OR drug* OR agent* OR therap* | 1,085,065 |
| 4 | “chronic disease*” OR diabet* OR “chronic lung disease*” OR “interstitial lung disease*” OR “pulmonary fibros*” OR COPD OR “chronic obstructive pulmonary disease*” OR asthm* OR “cardiovascular disease*” OR angina* OR hypertens* OR stroke* OR “cerebrovascular accident*” OR “heart disease*” OR “myocardial infarct*” OR arrhythm* OR “peripheral arter* disease*” OR “mental health” OR depress* OR anxiet* OR Parkinson* OR dementia* | 3,561,615 |
| 5 | 1 AND 2 AND 3 AND 4 | 140 |

Table F. Search strategies (PubMed)

| Steps | Search terms | Results (August 29, 2025) |
| --- | --- | --- |
| 1 | “artificial intelligence” OR AI OR “AI agent*” OR “artificial agent*” OR “assistance technolog*” OR “bot” OR “chat bot*” OR chatbot* OR chatterbot* OR chatgpt OR “ChatGPT” OR “GPT” OR “conversational agent*” OR “conversational AI” OR “conversational assistant*” OR “voice agent*” OR “conversational bot*” OR “conversational interface*” OR “conversational system*” OR “dialog system*” OR “dialogue agent*” OR “digital assistant*” OR “digital coach” OR “virtual coach” OR “embodied conversational agent*” OR “large language model*” OR LLM | 1,339,131 |
| 2 | adheren* OR complian* OR persist* OR concordan* OR continu* OR dropout* | 2,718,446 |
| 3 | medicat* OR pharma* OR drug* OR agent* OR therap* | 14,053,775 |
| 4 | “chronic disease*” OR diabet* OR “chronic lung disease*” OR “interstitial lung disease*” OR “pulmonary fibros*” OR COPD OR “chronic obstructive pulmonary disease*” OR asthm* OR “cardiovascular disease*” OR angina* OR hypertens* OR stroke* OR “cerebrovascular accident*” OR “heart disease*” OR “myocardial infarct*” OR arrhythm* OR “peripheral arter* disease*” OR “mental health” OR depress* OR anxiet* OR Parkinson* OR dementia* | 1,259,306 |
| 5 | 1 AND 2 AND 3 AND 4 | 1,634 |

Table G. Search strategies (Cochrane Library)

| Steps | Search terms | Results (August 29, 2025) |
| --- | --- | --- |
| 1 | artificial NEXT intelligence OR AI OR AI NEXT agent* OR artificial NEXT agent* OR assistance NEXT technolog* OR bot OR chat NEXT bot* OR chatbot* OR chatterbot* OR chatgpt OR ChatGPT OR GPT OR conversational NEXT agent* OR conversational NEXT AI OR conversational NEXT assistant* OR voice NEXT agent* OR conversational NEXT bot* OR conversational NEXT interface* OR conversational NEXT system* OR dialog NEXT system* OR dialogue NEXT agent* OR digital NEXT assistant* OR digital NEXT coach OR virtual NEXT coach OR embodied NEXT conversational NEXT agent* OR large NEXT language NEXT model* OR LLM | 13,733 |
| 2 | adheren* OR complian* OR persist* OR concordan* OR continu* OR dropout* | 328,080 |
| 3 | medicat* OR pharma* OR drug* OR agent* OR therap* | 1,299,721 |
| 4 | chronic NEXT disease* OR diabet* OR chronic NEXT lung NEXT disease* OR interstitial NEXT lung NEXT disease* OR pulmonary NEXT fibros* OR COPD OR chronic NEXT obstructive NEXT pulmonary NEXT disease* OR asthm*  OR cardiovascular NEXT disease* OR angina* OR hypertens* OR stroke* OR cerebrovascular NEXT accident* OR heart NEXT disease* OR myocardial NEXT infarct* OR arrhythm* OR peripheral NEXT arter* NEXT disease* OR mental NEXT health OR depress* OR anxiet* OR Parkinson* OR dementia* | 593,371 |
| 5 | 1 AND 2 AND 3 AND 4 | 742 |

Table H. Search strategies (CINAHL)

| Steps | Search terms | Results (August 29, 2025) |
| --- | --- | --- |
| 1 | “artificial intelligence” OR AI OR “AI agent*” OR “artificial agent*” OR “assistance technolog*” OR “bot” OR “chat bot*” OR chatbot* OR chatterbot* OR chatgpt OR “ChatGPT” OR “GPT” OR “conversational agent*” OR “conversational AI” OR “conversational assistant*” OR “voice agent*” OR “conversational bot*” OR “conversational interface*” OR “conversational system*” OR “dialog system*” OR “dialogue agent*” OR “digital assistant*” OR “digital coach” OR “virtual coach” OR “embodied conversational agent*” OR “large language model*” OR LLM | 17,504 |
| 2 | adheren* OR complian* OR persist* OR concordan* OR continu* OR dropout* | 254,902 |
| 3 | medicat* OR pharma* OR drug* OR agent* OR therap* | 822,130 |
| 4 | “chronic disease*” OR diabet* OR “chronic lung disease*” OR “interstitial lung disease*” OR “pulmonary fibros*” OR COPD OR “chronic obstructive pulmonary disease*” OR asthm* OR “cardiovascular disease*” OR angina* OR hypertens* OR stroke* OR “cerebrovascular accident*” OR “heart disease*” OR “myocardial infarct*” OR arrhythm* OR “peripheral arter* disease*” OR “mental health” OR depress* OR anxiet* OR Parkinson* OR dementia* | 471,728 |
| 5 | 1 AND 2 AND 3 AND 4 | 155 |

Table I. Search strategies (APA PsycArticle)

| Steps | Search terms | Results (August 29, 2025) |
| --- | --- | --- |
| 1 | “artificial intelligence” OR AI OR “AI agent*” OR “artificial agent*” OR “assistance technolog*” OR “bot” OR “chat bot*” OR chatbot* OR chatterbot* OR chatgpt OR “ChatGPT” OR “GPT” OR “conversational agent*” OR “conversational AI” OR “conversational assistant*” OR “voice agent*” OR “conversational bot*” OR “conversational interface*” OR “conversational system*” OR “dialog system*” OR “dialogue agent*” OR “digital assistant*” OR “digital coach” OR “virtual coach” OR “embodied conversational agent*” OR “large language model*” OR LLM | 3,865 |
| 2 | adheren* OR complian* OR persist* OR concordan* OR continu* OR dropout* | 125,556 |
| 3 | medicat* OR pharma* OR drug* OR agent* OR therap* | 79,984 |
| 4 | “chronic disease*” OR diabet* OR “chronic lung disease*” OR “interstitial lung disease*” OR “pulmonary fibros*” OR COPD OR “chronic obstructive pulmonary disease*” OR asthm* OR “cardiovascular disease*” OR angina* OR hypertens* OR stroke* OR “cerebrovascular accident*” OR “heart disease*” OR “myocardial infarct*” OR arrhythm* OR “peripheral arter* disease*” OR “mental health” OR depress* OR anxiet* OR Parkinson* OR dementia* | 193,660 |
| 5 | 1 AND 2 AND 3 AND 4 | 1,505 |
