# Supplementary material for: Artificial intelligence–based chatbots to enhance medication adherence among patients with non-communicable chronic diseases: Systematic review and meta-analysis
Source: PLOS Digit Health. 2026 Jul 16;5(7):e0001507. doi: 10.1371/journal.pdig.0001507 (PMC13375028; doi:10.1371/journal.pdig.0001507)
Supplement: S3 Appendix — (DOCX) [file pdig.0001507.s003.docx]

**S3 Appendix. Quality assessment for RCTs and single-arm pre-post studies**

Table A. Quality assessment of RCTs (assessed by RoB2)

| No. | Ref | Randomization Process | Deviations from the intended interventions | Missing outcome data | Measurement of the outcome | Selection of the reported result | Overall risk-of-bias judgement |
| --- | --- | --- | --- | --- | --- | --- | --- |
| 1 | Labovitz et al., 2017 | Low | Low | Low | Low | Low | Low |
| 2 | Horne et al., 2022 | Low | Low | Low | Low | Low | Low |
| 3 | Prakash et al., 2024 | Low | Low | Low | Low | Low | Low |
| 4 | Arshed et al., 2024 | Low | Low | Low | Low | Low | Low |
| 5 | Caballero Mateos et al., 2025 | Low | Low | Low | Low | Low | Low |
| 6 | Ho et al., 2025 | Low | Low | Low | Low | Low | Low |

Table B. Quality assessment of single-arm pre-post studies (by ROBINS-E)

| No. | Ref | Confounding | Measurement of exposure | Selection of participants into the study | Post-exposure interventions | Missing data | Measurement of outcomes | Selection of the reported result | Overall risk-of-bias judgement |
| --- | --- | --- | --- | --- | --- | --- | --- | --- | --- |
| 1 | Lau-Min et al, 2024 | Low | Low | Low | Low | Low | Low | Low | Low |

Table C. Details of quality assessment of RCT included in the meta-analysis (assessed by RoB2)

|  | Labovitz et al., 2017 | Horne et al., 2022 | Prakash et al., 2024 | Arshed et al., 2024 | Caballero Mateos et al., 2025 | Ho et al., 2025 |
| --- | --- | --- | --- | --- | --- | --- |
| **1. Randomization process** |  |  |  |  |  |  |
| 1.1 Was the allocation sequence random? | Yes | Yes | Yes | Yes | Yes | Yes |
| 1.2 Was the allocation sequence concealed until participants were enrolled and assigned to the intervention? | Yes | Yes | Yes | Yes | Yes | Yes |
| 1.3 Did baseline difference between intervention groups suggest a problem with the randomization process? | No | No | No | No | No | No |
| Overall judgement for randomization process | Low risk | Low risk | Low risk | Low risk | Low risk | Low risk |
| **2. Deviations from the intended interventions** |  |  |  |  |  |  |
| 2.1 Were participants aware of their assigned intervention during the trial? | Yes, the control group received no intervention | Yes, the control group received usual care for cardiovascular diseases | Yes, the control group received no intervention | Yes, the control group received usual care for hypertension | Yes, the control group received usual care for type 2 diabetes | Yes, the control group received no intervention |
| 2.2 Were people delivering the interventions were aware of participants’ assigned intervention during the trial? | No, pill counts (objective outcome is less likely to be affected by lack of blinding) | No, proportion of days covered was measured objectively from pharmacy claims data, which were less likely to be affected by lack of blinding | Yes, Medication Adherence Measure Scale was self-reported | Yes, medication adherence was measured by self-reported pill counts and validated SEAMS questionnaire. | Yes, medication adherence was measured by 4-item Morisky–Green–Levine Medication Adherence Questionnaire | No, medication adherence were measured objectively by Proportion of days covered over 12 months that was less likely to be affected by lack of blinding |
| 2.3 Were the deviations from the intended intervention that arose because of the trial context? | No, all participants received assigned interventions | No, all participants received assigned interventions | No, all participants received assigned interventions | No, all participants received assigned interventions | No, all participants received assigned interventions | No, all participants received assigned interventions |
| 2.4 Were these deviations likely to affect the outcome? | N.A. | N.A. | N.A. | N.A. | N.A. | N.A. |
| 2.5 Were these deviations from intended intervention balanced between groups? | N.A. | N.A. | N.A. | N.A. | N.A. | N.A. |
| 2.6 Was an appropriate analysis used to estimate the effect of assignment to intervention? | Yes, intention-to-treat analysis was used | Yes, intention-to-treat analysis was used | Yes, intention-to-treat analysis was used | Yes, intention-to-treat analysis was used | Yes, intention-to-treat analysis was used | Yes, intention-to-treat analysis was used |
| 2.7 Was there potential for a substantial impact of the failure to analyze participants in the group to which they were randomized? | N.A. | N.A. | N.A. | N.A. | N.A. | N.A. |
| Overall judgement for deviations from the intended interventions | Low risk | Low risk | Low risk | Low risk | Low risk | Low risk |
| **3. Risk of bias due to missing outcome data** |  |  |  |  |  |  |
| 3.1 Were data of this outcome available for all, or nearly all, participants randomized? | Yes, all patients in the intervention group and 1 out of 13 patients in the control group had complete data | No, 4 out of 93 patients in the intervention group, and all patients in the control group had complete data | No information about that | No, 6 out of 220 participants in the intervention group, and 10 out of 219 participants in the control group had complete data | Yes, all participants had complete data | No, 56 out of 2375 participants in the intervention group, and 55 out of 2376 participants in the control group had complete data |
| 3.2 Is there evidence that the results was not biased by missing outcome data? | N.A. | No, the study excluded dropouts or those without complete data from the analysis | N.A. | Probably no, missing data were reported and treated using the single  imputation approach | N.A. | Probably not, to account for potential data missing at  random, observation-specific weights specific to each  person-period were calculated using logistic regression to  estimate the inverse probability that a longitudinal value  was observed. |
| 3.3 Could missing in the outcome depend on its true value? | N.A. | Probably no, documented reasons of missing outcome data were unrelated to the outcome | N.A. | Probably no, documented reasons of missing outcome data were unrelated to the outcome | N.A. | Probably no, documented reasons of missing outcome data were unrelated to the outcome |
| 3.4 Is it likely that missing in outcome depended on its true value? | N.A. | Probably no, proportion and reported reasons of missing data was comparable between groups, and reported reasons of missing outcome data were unrelated to the outcomes | N.A. | Probably no, proportion and reported reasons of missing data was comparable between groups, and reported reasons of missing outcome data were unrelated to the outcomes | N.A. | Probably no, proportion and reported reasons of missing data was comparable between groups, and reported reasons of missing outcome data were unrelated to the outcomes |
| Overall judgement for missing outcome | Low risk | Low risk | Low risk | Low risk | Low risk | Low risk |
| **4. Risk of bias in measurement of the outcome** |  |  |  |  |  |  |
| 4.1 Was the method of measuring the outcome inappropriate? | No, pill counts was measured objectively | No, proportion of days covered was measured objectively from pharmacy claims data | No, medication adherence was measured by Medication Adherence Measure Scale | No, medication adherence were measured by pill counts and validated SEAMS questionnaire. SEAMS is a validated  and reliable questionnaire. | No, medication adherence was measured by 4-item Morisky–Green–Levine Medication. This scale has a total score of 0 to 4 points. A score of 4 denotes  high adherenc | No, medication adherence were measured objectively by Proportion of days covered over 12 |
| 4.2 Could measurement or ascertainment of the outcome between intervention group? | No, same measurements for both groups | No, same measurements for both groups | No, same measurements for both groups | No, same measurements for both groups | No, same measurements for both groups | No, same measurements for both groups |
| 4.3 Were outcome assessors aware of the intervention received by study participants? | No, outcome assessors were blinded | No, outcome assessors were blinded | No, outcome assessors were blinded | No, outcome assessors were blinded | No, outcome assessors were blinded | No, outcome assessors were blinded |
| 4.4 Could assessment of the outcome have been influenced by knowledge of intervention received? | N.A. | N.A. | N.A. | N.A. | N.A. | N.A. |
| 4.5 Is it likely the assessment of the outcome was influenced by knowledge of intervention received? | N.A. | N.A. | N.A. | N.A. | N.A. | N.A. |
| Overall judgement for measurement of the outcome | Low risk | Low risk | Low risk | Low risk | Low risk | Low risk |
| **5. Risk of bias in selection of the reported result** |  |  |  |  |  |  |
| 5.1 Were the data that produced this result analyzed in accordance with a pre-specified analysis plan that was finalized before unblended outcome data were available for analysis? | Yes, the results were analyzed in line with a published protocol | Yes, the results were analyzed in line with a published protocol | Yes, the results were analyzed in line with a registered analytical plan | Yes, the results were analyzed in line with a registered analytical plan | Yes, the results were analyzed in line with a registered analytical plan | Yes, the results were analyzed in line with the protocol |
| 5.2 Are the results being assessed likely to have been selected from multiple eligible outcome measurements? | No | No | No | No | No | No |
| 5.3 Are the results being assessed likely to have been selected from multiple eligible analyses of the data? | No | No | No | No | No | No |
| Overall judgement in selection of the reported results | Low risk | Low risk | Low risk | Low risk | Low risk | Low risk |
| **Overall risk of bias judgement** | Low risk | Low risk | Low risk | Low risk | Low risk | Low risk |
