# Supplementary material for: Artificial intelligence–based chatbots to enhance medication adherence among patients with non-communicable chronic diseases: Systematic review and meta-analysis
Source: PLOS Digit Health. 2026 Jul 16;5(7):e0001507. doi: 10.1371/journal.pdig.0001507 (PMC13375028; doi:10.1371/journal.pdig.0001507)
Supplement: S4 Appendix — (OCX) [file pdig.0001507.s004.docx]

**S4 Appendix. Forest plots for sub-group analyses
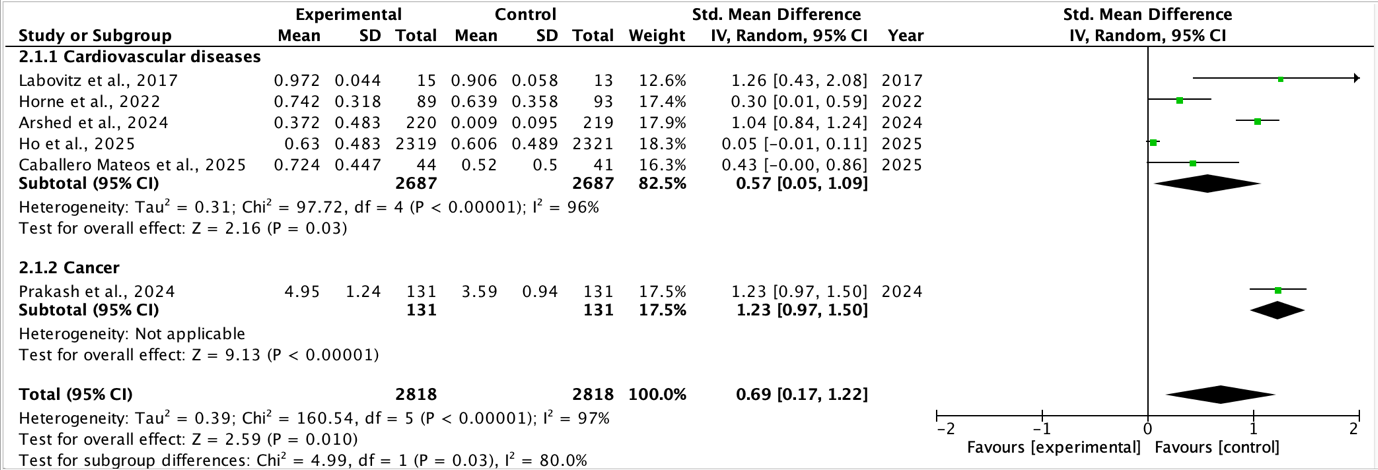
**

Fig A. Forest plot of included studies stratified by disease type


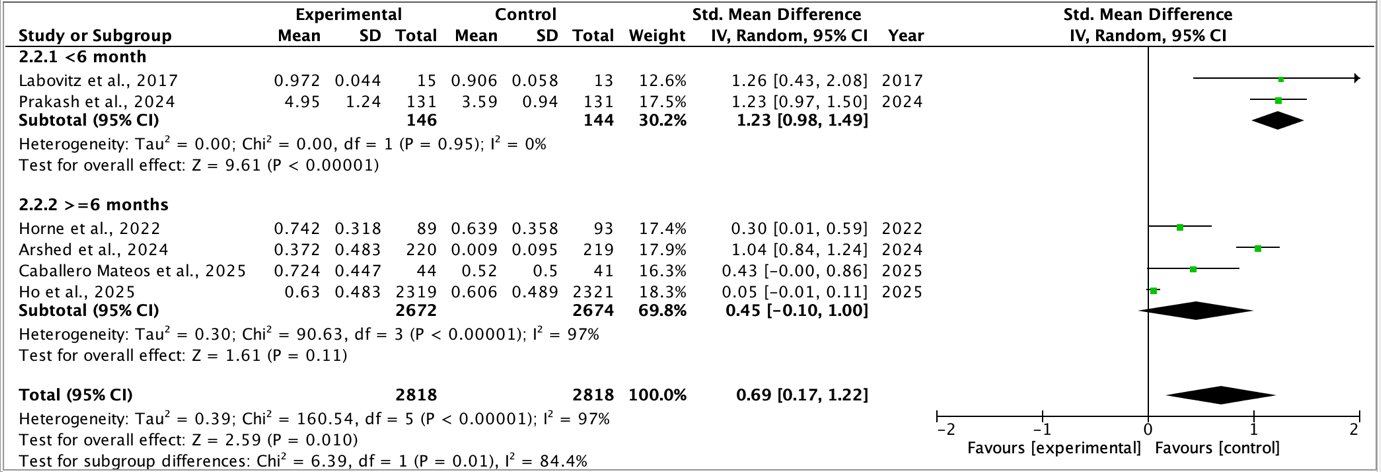


Fig B. Forest plot of included studies stratified by intervention duration


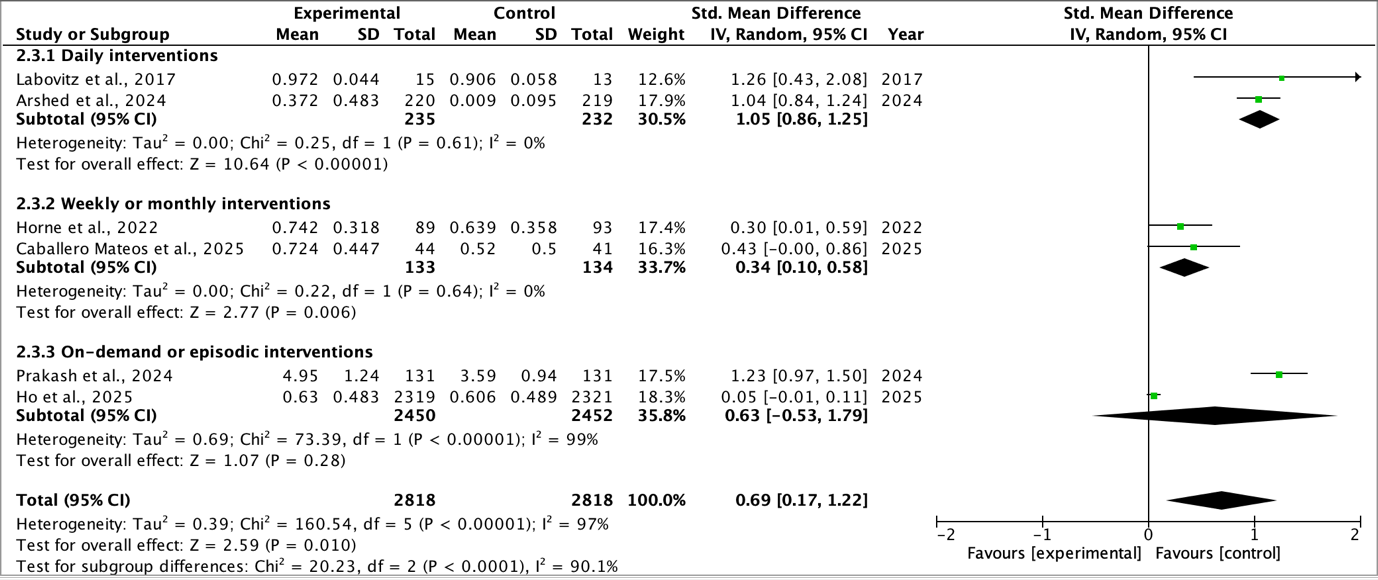


Fig C. Forest plot of included studies stratified by intervention intensity


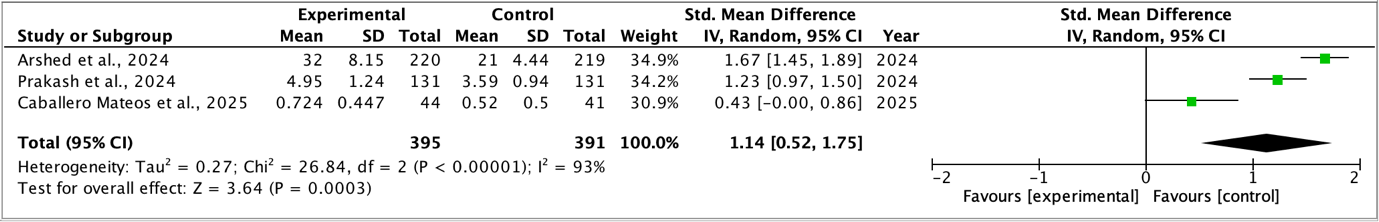


Fig D. Forest plot of included studies stratified by self-reported outcomes


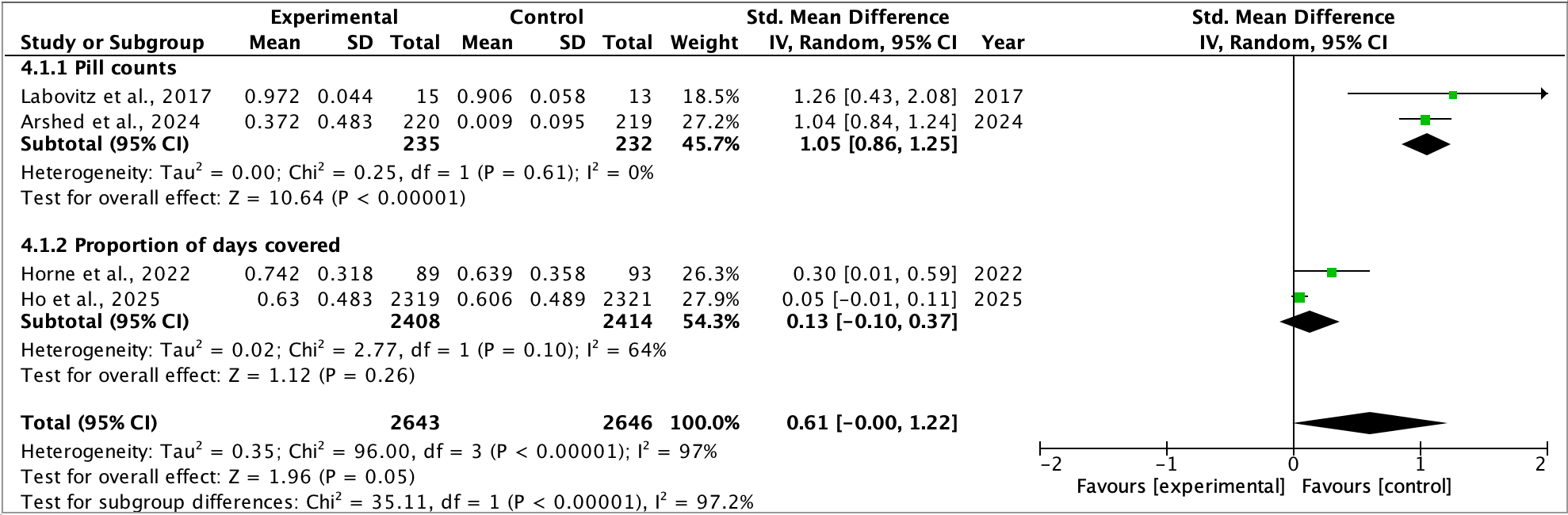


Fig E. Forest plot of included studies stratified by objective outcomes


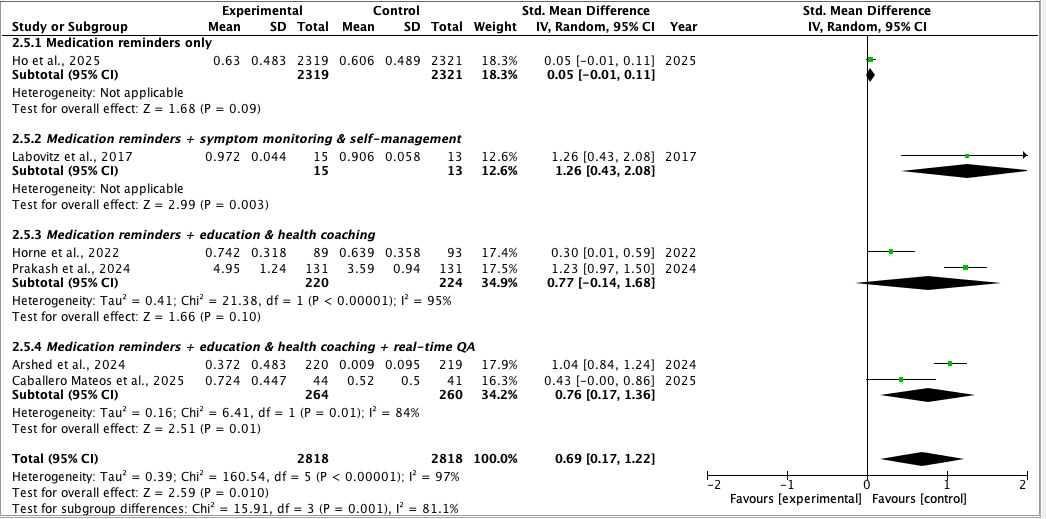


Fig F. Forest plot of included studies stratified by chatbot functions
