# Supplementary material for: Artificial intelligence–based chatbots to enhance medication adherence among patients with non-communicable chronic diseases: Systematic review and meta-analysis
Source: PLOS Digit Health. 2026 Jul 16;5(7):e0001507. doi: 10.1371/journal.pdig.0001507 (PMC13375028; doi:10.1371/journal.pdig.0001507)
Supplement: S5 Appendix — (DOCX) [file pdig.0001507.s005.docx]

**S5 Appendix. Funnel plots**


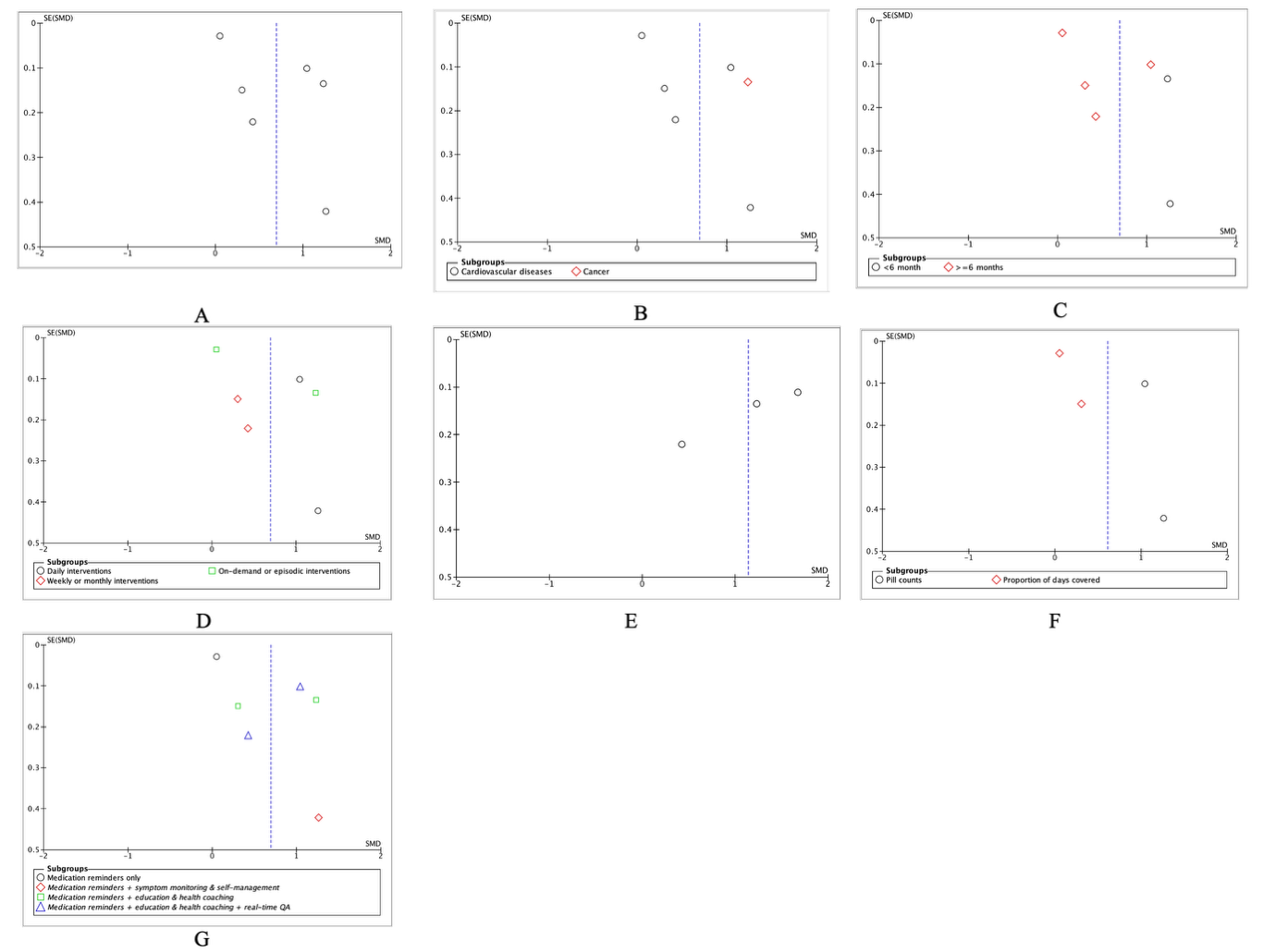


**Fig A. Funnel plots assessing potential publication bias among** included studies**.**
(A) Funnel plot for all included studies; (B) funnel plot stratified by disease type; (C) funnel plot stratified by intervention duration; (D) funnel plot stratified by intervention intensity; (E) funnel plot stratified by self-reported outcomes; (F) funnel plot stratified by objective outcomes; (G) funnel plot stratified by chatbot functions.
